# Partial structure, dampened mobility, and modest impact of a His tag in the SARS-CoV-2 Nsp2 C-terminal region

**DOI:** 10.1007/s00249-021-01575-9

**Published:** 2021-10-11

**Authors:** Miguel Mompeán, Miguel Á. Treviño, Douglas V. Laurents

**Affiliations:** “Rocasolano” Institute for Physical Chemistry, Spanish National Research Council, Serrano 119, 28006 Madrid, Spain

**Keywords:** SARS-CoV-2, Nsp2, NMR spectroscopy, Intrinsically disordered proteins

## Abstract

Intrinsically disordered proteins (IDPs) play essential roles in regulating physiological processes in eukaryotic cells. Many viruses use their own IDPs to “hack” these processes to deactivate host defenses and promote viral growth. Thus, viral IDPs are attractive drug targets. While IDPs are hard to study by X-ray crystallography or cryo-EM, atomic level information on their conformational preferences and dynamics can be obtained using NMR spectroscopy. SARS-CoV-2 Nsp2, whose C-terminal region (CtR) is predicted to be disordered, interacts with human proteins that regulate translation initiation and endosome vesicle sorting. Molecules that block these interactions could be valuable leads for drug development. The ^13^Cβ and backbone ^13^CO, ^1^HN, ^13^Cα, and ^15^N nuclei of Nsp2’s 45-residue CtR were assigned and used to characterize its structure and dynamics in three contexts; namely: (1) retaining an N-terminal His tag, (2) without the His tag and with an adventitious internal cleavage, and (3) lacking both the His tag and the internal cleavage. Two five-residue segments adopting a minor extended population were identified. Overall, the dynamic behavior is midway between a completely rigid and a fully flexible chain. Whereas the presence of an N-terminal His tag and internal cleavage stiffen and loosen, respectively, neighboring residues, they do not affect the tendency of two regions to populate extended conformations.

**Supplementary Information:**

The online version contains supplementary material available at 10.1007/s00249-021-01575-9.

## Introduction

The pandemic provoked by SARS-CoV-2 is sparking unprecedented efforts in the development of vaccines. Nevertheless, drugs which block the activity of viral proteins should also be sought, since SARS-CoV-2 vaccines may well lack availability and complete efficacy, particularly for the immune compromised or against novel strains. Intrinsically disordered proteins (IDPs) play essential roles in regulating physiological processes in eukaryotic cells by forming weak protein/protein interactions. Many viruses use their own IDPs to trick these processes to neutralize host defenses and promote viral growth (Davey et al. 2011). For example, the small HIV protein Tat (Transactivator of Transcription) is intrinsically disordered (Shojania and O’Neil 2006) and is key to express viral genes; in addition, it manipulates cell signalling networks to downregulate apoptosis and alters cytokine expression (Clark et al. 2016). Because of their key functions, viral IDPs, such as HIV Tat (Hamy et al. 2000), are attractive drug targets. While the flexibility of IDPs generally precludes their study by X-ray crystallography or cryo-EM, atomic level information on their conformational preferences and dynamics can be obtained using NMR spectroscopy.

Using bioinformatics tools, a C-terminal region of SARS-CoV-2 Nsp2 has been predicted to be disordered (Giri et al*.* 2021). Interestingly, this 45-residue Nsp2 region appears to be more disordered in SARS-CoV-2 than in its close homologs human SARS (responsible for the 2003 outbreak) and bat coronavirus (see Sup. Figure 9 in Giri et al*.* 2021). A study (Gordon et al. 2020a) of the SARS-CoV-2 interactome reported that Nsp2 interacts with human proteins GIGYF2 and EIF4E2, which regulate translation initiation, as well as WASHC4 and FKBP15, which are involved in endosome vesicle sorting. This investigation has been recently extended by comparative analyses with SARS-CoV-1 and MERS-CoV interactomes and implicated Nsp2 in exosomes, cellular respiration, lipid transport (Gordon et al. 2020b). Nsp2 from human SARS was also reported to interact with Prohibitin1/2, a cell proliferation modulator with tumor suppression activity (Cornilley-Ty et al*.* 2009). Molecules that block these interactions could be valuable leads for drug development.

Some months after a preprint of this study was made public (Mompeán et al. 2020), a preliminary study was posted on BioRxiv which reported a medium resolution structural model of the complete SARS CoV-2 Nsp2 protein based on CryoEM and AI (Gupta et al. 2021). In their report, electron density for the C-terminal region was either missing (which strongly suggests disorder) or indicated a folded domain rich in β-structure depending on the conditions. Based on analysis of Nsp2 mutants, Gupta et al. (2021) also proposed that E63, E65, G262, G265, K330, and K337 are key for binding host proteins. No Nsp2 C-terminal residues/host protein interactions have yet been identified. The main objective of this study is to characterize the conformational preferences and dynamics of the putatively disordered C-terminal region of SARS CoV-2 Nsp2 (Nsp2 CtDR) using NMR spectroscopy. The assignments obtained could also guide future studies of interactions between Nsp2, human target proteins, and inhibitors.

Finally, His tags are a popular tool for modern protein purification. They are usually removed after the first purification step, but sometimes they are occluded and difficult to remove. His tags left on folded proteins have been reported to influence biochemical activity (Majorek et al. 2014) or increase protein rigidity (Thielges et al. 2011), but their effects on disordered proteins are less well characterized. To address this knowledge gap, we also report the NMR characterization of an Nsp2 CtDR construct with an N-terminal His tag. The secondary structure and dynamics of an additional third sample without the His tag but that had suffered an internal cleavage, possibly due to enterokinase, are also reported.

## Results

### Bioinformatics analysis

**Fig. 1:**
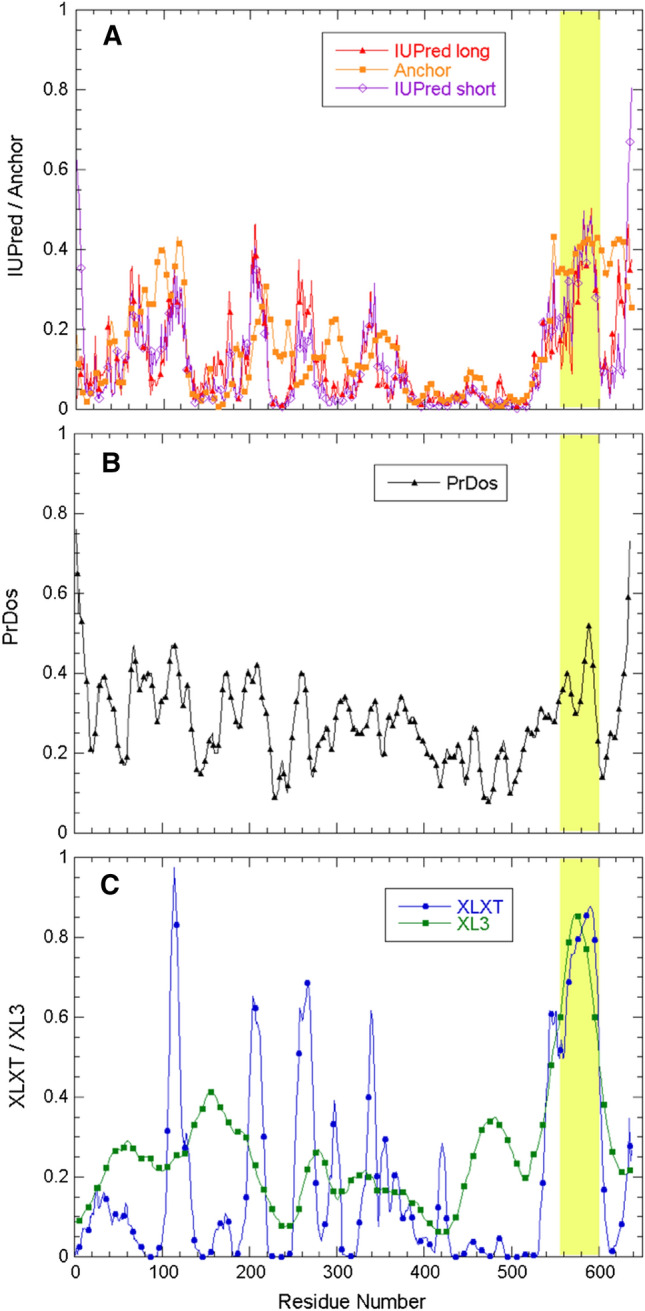
SARS-CoV-2 Nsp2. Predicted disordered region and primary structure. **A** Sequence-based disorder tendency scores for Nsp2 calculated with IUPred and Anchor, **B** PrDos, and **C** PONDR^®^ XLXT and XL3. The region studied here is highlighted in light yellow. **D** Nsp2 primary structure (severe acute respiratory syndrome coronavirus 2). NCBI reference sequence: YP_009742609.1

The C-terminal region of Nsp2 was recently predicted to be disordered by Giri et al*.* (2021), who applied the PONDR^®^ and IUPRED algorithms. Nevertheless, this region contains a rather high proportion of hydrophobic residues which generally promote order and folding; in particular its content of Phe, Ile, Leu, Met, Tyr, Val, and Trp residues is 33.3%, which is close to their average content in folded proteins (32.2%) and much higher than their average content in IDPs (22.0%) (Uversky 2013). To corroborate these findings, we have applied the same tools as Giri et al*.* (2021) as well as the PrDOS disorder prediction algorithm (Ishida and Kinoshita 2007) to the Nsp2 sequence (Fig. 1). Whereas the results show that the C-terminal region spanning approximately residues 555–600 have a high disorder propensity as compared to the rest of the protein, the score falls short of the threshold for some algorithms used by Giri et al*.* (2021) and is close to the PrDOS threshold for disorder (Fig. 1). Considering the distinct predictions of these bioinformatics tools, and the fact that disorder prediction programs are occasionally wrong (Treviño et al. 2018), we have used NMR spectroscopy to characterize structurally the putatively disordered region of Nsp2.

### NMR assignment and assessment of partial structure

The ^13^Cβ and backbone ^13^CO, ^1^HN, ^13^Cα, and ^15^N nuclei were assigned by analysis of a series of 2D ^1^H-^15^N HSQC and ^13^C-^15^ N CON as well as 3D HNCO, HNCA, CBCAcoNH, and HncocaNH (Pantoja-Uceda and Santoro 2009) spectra. The degree of completeness of the assignments is reported for the three samples studied are reported in Table 1 and assignments are deposited in the BMRB database under access code 50,687.

**Table 1:** NMR spectral parameters

Experiment			Number of scans		Sweep width (ppm)			Matrix
Assignment								
1D ^1^H			48		12			32 k
2D ^1^H-^15^N HSQC			4		11 ^1^H × 20 ^15^ N			2 k × 256
2D CON^a^			8		11 ^13^CO × 34 ^15^ N			1 k × 512
3D HNCO^a^			8		11 ^1^H × 20 ^15^ N × 12 ^13^C			2 k × 48 × 256
3D HNCA^a^			8		11 ^1^H × 20 ^15^ N × 25 ^13^Cα			2 K × 48 × 128
3D CBCAcoNH^a^			8		2 k ^1^H × 20 ^15^ N × 55 ^13^C			2 k × 48 × 128
3D HncocaNH			8		10 ^1^H × 20 ^15^ N × 10 ^1^H			2 k × 64 × 64
Relaxation								
2D HSQCTEFT3GPSI (T_1_rho)^b^			8		10 ^1^H × 20 ^15^ N			2 k × 384
2D HSQCNOEFGPSI {^1^H}-^15^N NOE^c^			16		10 ^1^H × 20 ^15^ N			2 k × 384
Completeness of NMR assignments								
|Sample/Nuclei- >		^13^Cα		^13^Cβ	^13^CO	^15^ N	^1^HN	
+ His tag		82%		82%	82%	92%	100%	
No His tag, cut		98%		87%	84%	98%	98%	
No His tag, not cut		100%		89%	93%	93%	98%	

^a^Recorded with non-uniform sampling. The % of sparse sampling was 35% for the 3D HNCO, HNCA, and CBCAcoNH experiments and 25% for the 2D CON experiment

^b^Delays used (in seconds): 0.008, 0.300, 0.036, 0.076, 0.900, 0.150, 0.500, 1.350

^c^Recycle delay = 10 s

**Fig. 2:**
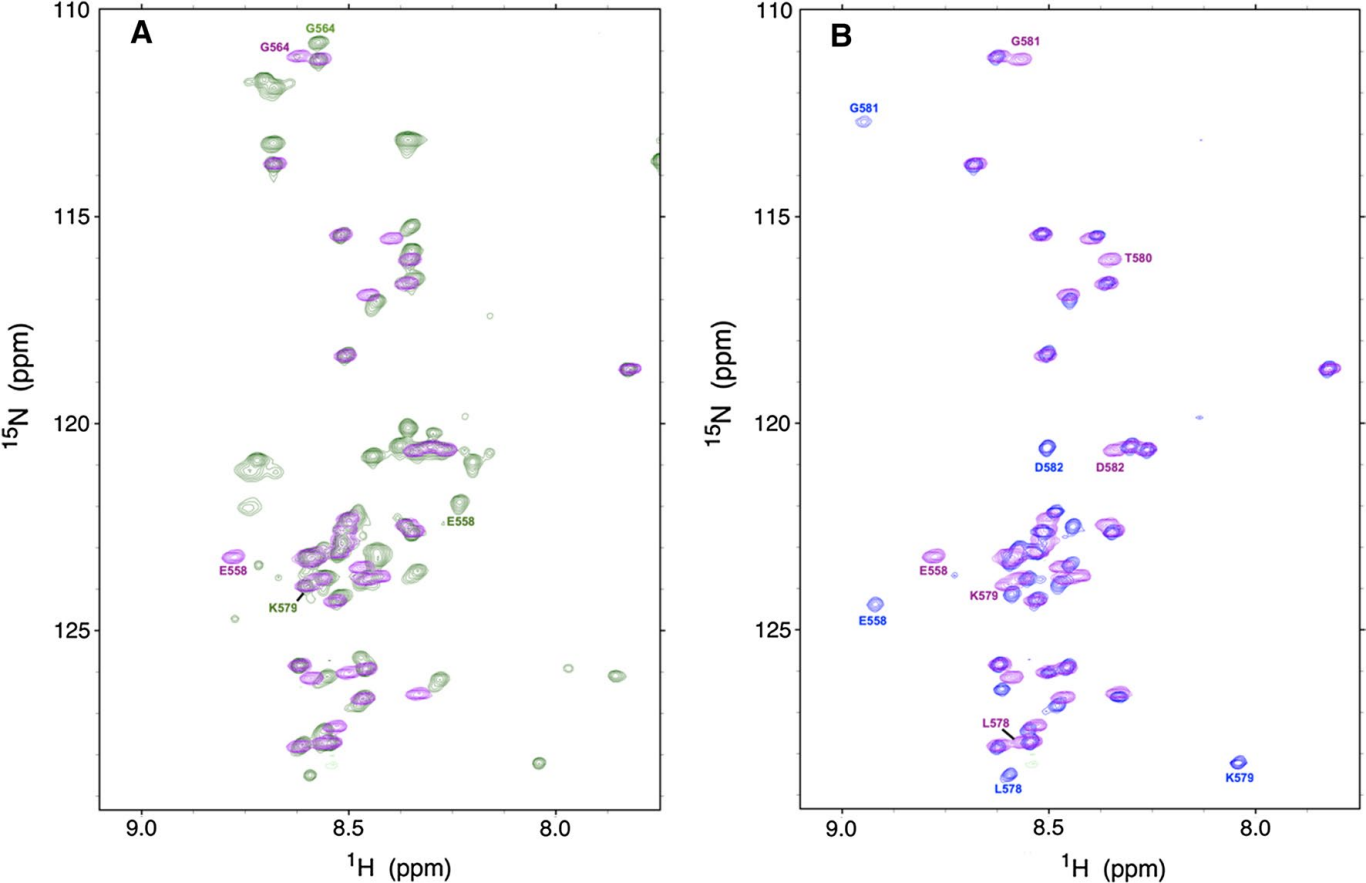
2D ^1^H-^15^N HSQC NMR spectra of Nsp2 CtDR in 5 mM KPi, 10 mM NaCl, pH 6.3, 5 °C. **A** Prior to cleavage of the His tag (green) and following removal of the His tag (purple). Peaks with distinct chemical shifts are labeled according to their numbering in the full length Nsp2 protein. Peaks arising from the N-terminal His tag are not labeled: The His tag sequence is: MAHHHHHHGTGTGSNDDDD-K. **B** Following His tag cleavage (purple) and with the region containing an additional break between K_579_ and T_580_ (blue). Peaks with the most relevant chemical shift changes are labeled. Each spectrum is shown separately and labeled in Sup. Fig. 1

**Fig. 3:**
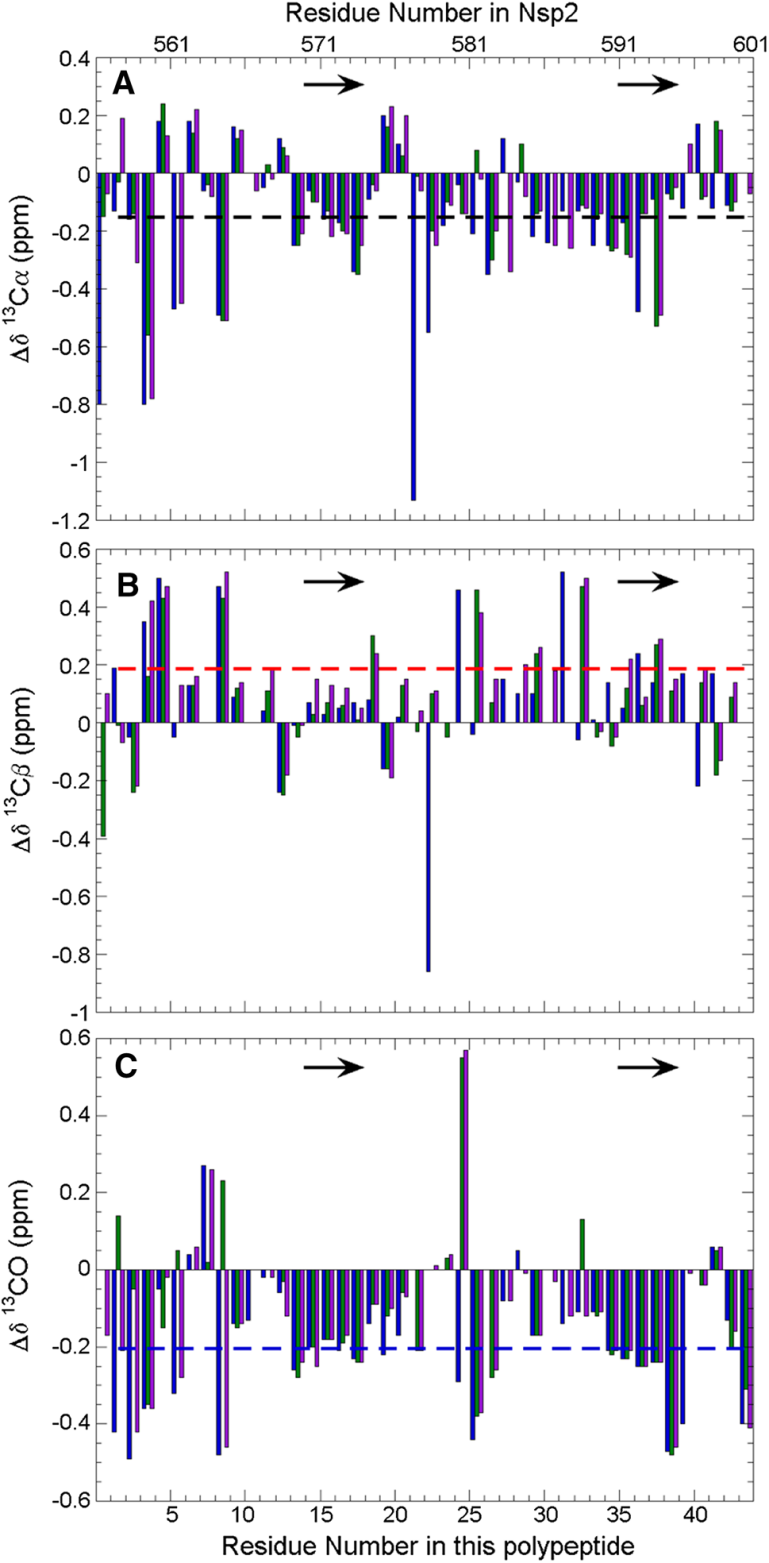
Conformational shift analysis for Nsp2 CtDR in 5 mM KPi, 10 mM NaCl, pH 6.3, 5 °C. Nsp2 CtDR conformational chemical shifts of **A** ^13^Cα (black), and **B** ^13^Cβ (red) and **C** ^13^CO (blue). Values for the Nsp2 CtDR prior to His tag cleavage (green bars), and following His tag cleavage with (blue) or without (purple) an additional cleavage between K_579_ and T_580_ are shown. The dashed black, red, and blue lines mark the values expected for 10% β-strand for ^13^Cα, ^13^Cβ, and ^13^CO, respectively. The position of two modestly populated β-strands are marked by arrows

The three assigned 2D ^1^H-^15^N HSQC spectra of the Nsp2 CtDR in the presence or absence of an N-terminal His tag and with or without an internal cleavage are shown in Fig. 2. The low ^1^HN signal dispersion observed in all three spectra suggests that the Nsp2 CtDR is chiefly disordered. The ^13^Cα, ^13^Cβ, and ^13^CO conformational chemical shifts (Δ*δ*) values are shown in Fig. 3. Overall, the Δ*δ* values are low, which evinces that the Nsp2 CtDR is mostly unfolded. However, two five-residue stretches spanning residues E_570_-VLTE_574_ and S_591_EAVE_595_ tend to adopt β-stands or extended conformations. Their populations within the conformational ensemble are about 10–11% for E_570_-VLTE_574_ and 12–14% for S_591_EAVE_595_. In addition, a few discontinuous residues at the N-terminus of this region also show some tendency to adopt β-strand or extended conformations (Fig. 3). It is important to point out that the sample whose His tag was cleaved contained an additional proteolytic cleavage between residues K_579_ and T_580_, which was identified on the basis of chemical shift alterations, the absence of an ^1^H-^15^N crosspeak for T_580_ and the relaxation measurements reported below. This break occurs between the two partly ordered segments mentioned above. Since this break would disrupt hydrogen bonding and stabilizing contacts between the two extended conformations if they were to adopt interacting β-strands, and since populations for E_570_–E_574_ and S_591_–E_595_ are similar in both samples, this strongly suggests that the conformational chemical shifts arise from extended conformations instead of β-rich secondary structure.

### Dynamics

**Fig. 4:**
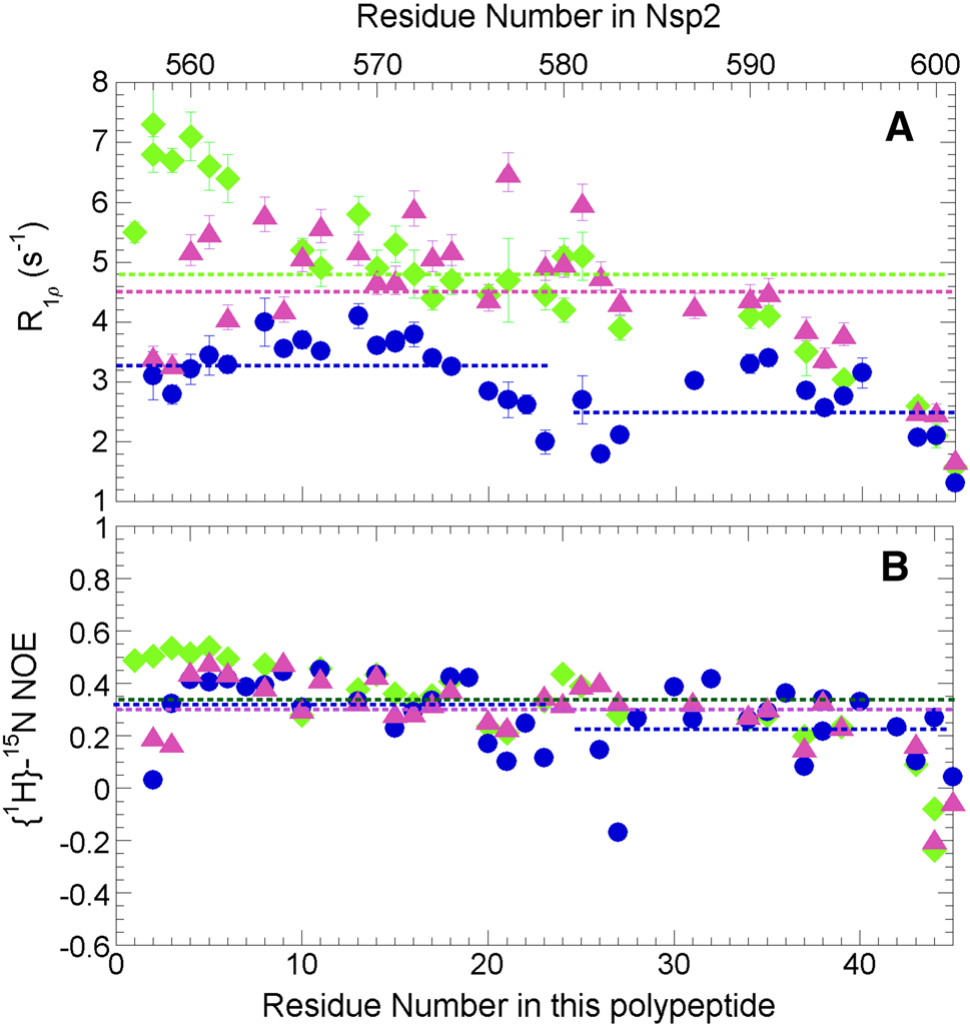
Residue level dynamics from ^15^N relaxation. **A** Relaxation rates in the rotating frame (R_1_ρ) for the Nsp2 CtDR with (green) the His tag present, with the His tag and with a cleavage after residue K_579_ (blue) and without the His tag and without the cleavage (purple). In this panel, as well as **B** the mean values for each sample, and both fragments of the cut sample, are represented by the dashed lines with the same green, blue, purple color code. **B** {^1^H}-^15^N NOE ratio values for the Nsp2 CtDR with (green) the His tag present, with the His tag and with a cleavage after residue K_579_ (blue) and without the His tag and without the cleavage (purple). As the calculated errors are less than 0.02, no error bars are shown as they are smaller than the symbols. The hNOE ratio values are less than values usually seen in folded proteins’ rigid regions (0.70–0.80) or flexible zones (0.60–0.65) but higher than values typical of highly flexible segments (≤ 0)

To assess the extent and contributions of internal motions to the dynamics, we measured relaxation rates in the rotating time frame (R_1_ρ) for the Nsp2 CtDR. This experiment is sensitive to both fast (ps-ns) and slow (µs-ms) internal motions arising from exchange. Generally low values are found, reflecting flexibility and fast dynamics in the ps-ns timescale. A closer inspection reveals that the N-terminal half of the sequence features increased R_1_ρ relaxation rates that decrease towards the C-terminus. The higher values in the N-terminus indicate dampened mobility of this region, likely due to conformational exchange in the µs-ms timescale for this segment. In contrast, unrestricted, fast dynamics are observed towards the C-terminus, with gradually decreasing R_1_ρ relaxation rates (Fig. 4A). The presence of the His tag seems to further stiffen the N-terminal region of the polypeptide. However, beyond the first ten residues, its effect is negligible and the same dynamic behavior is observed, with decreasing R_1_ρ relaxation rates towards the C-terminus that feature similar relaxation rate values. These results together suggest that the N-terminal part exhibits dampened mobility, more so when extended upstream by the His tag, and that the mobility is progressively less restricted towards the C-terminus. By contrast, the presence of an internal cleavage that yields two fragments has a dramatic effect on this trend, and results in two fragments that show faster internal motions. In particular, the average R_1ρ_ rates decrease from 4.5 (no His tag) to 3.3 s^−1^ and 2.6 s^−1^ (no His tag, but internally cleaved, N- and C-ter fragments, respectively) (Fig. 4A). These results are point to a length dependence of the potential conformational exchange that underlies restricted mobility, i.e. higher R_1_ρ rates, in the N-terminus of uncleaved constructs.

On fast ps-ns timescales, the {^1^H}-^15^N NOE measurements show that the presence of the N-terminal His tag also appears to increase the rigidity of the polypeptide in the first several residues; thereafter, it does not seem to alter the dynamics significantly (Fig. 4B). The presence of the cleavage moderately increases the flexibility in resulting C-terminal fragment, however, on this timescale, the break does not substantially affect the dynamics of residues of the resulting N-terminal fragment (Fig. 4B). Overall the {^1^H}-^15^N NOE ratios range between 0.2 and 0.4, except for residues near termini which show lower values (Fig. 4B). Whereas these values are lower than typical ratios seen in the rigid elements of folded proteins (0.7–0.8), they are significantly higher than those observed in some other IDPs such as α-synuclein (Masaracchia et al. 2020).

## Discussion

Despite recent findings that SARS-CoV-2 Nsp2 is undergoing positive nature selection and is thus important to the virus (Flores-Alanis et al. 2021), this protein is a relatively uncharacterized. Here, we show that the C-terminal region of SARS-CoV-2 Nsp2 is intrinsically disordered, which is in general agreement with bioinformatics predictions (Giri et al*.* 2021). Nevertheless, two five-residue stretches show a small, but significant tendency to adopt extended/β-strand conformations, and the region is more rigid than a completely disordered protein chain. These findings may be attributed to the high content of β-branched or bulky residues as well as a high density of negatively charged residues which favored extended or β-conformations (Minor and Kim 1994; Zhou and Pang 2018). These β-strands may be present in the folded form of the Nsp2 C-terminal region in a recently advanced structural model (Gupta et al. 2021), although a direct comparison is not possible since no residue level information on this region could be discerned from this medium (5–6 Å) resolution model. These results seem not to depend on the presence of an N-terminal His tag or an internal proteolytic cleavage, which suggests these extended conformations may form independently. Hypothetically, the C-terminal region, which appears to be rather autonomous with respect to the rest of the protein structure (Gupta et al. 2021) might interact electrostatically with the second and third domains, which are rich in positively charged residues.

Nsp2 has been found to interact with many human proteins and functional complexes (Cornillez-Ty et al 2009; Gordon et al 2020a, b) and some residues in its N-terminal domains have already been implicated in these interactions (Gupta et al. 2021). The Nsp2 CtDR might also participate in interactions with human proteins, as suggested by the relatively high ANCHOR score (Fig. 1) and future experiments will test whether their degree of structure and rigidity increase upon binding. Blocking such an interaction might be an excellent target for future pharmaceutics. Inhibitor screening of the Nsp2 CtDR in collaboration with the COVID19-NMR consortium is underway. The assigned ^1^H-^15^N spectrum could serve in the near future to identify where first-generation inhibitors bind and thereby help guide their improvement.

His tags are a common tool for facilitating recombinant protein purification and they are usually removed prior to conformational characterization. The results presented here suggest that this removal may not always be necessary for disordered polypeptides poor in anionic residues since its effect on protein conformation and dynamics is limited to the first neighboring residues. Here, the His tag may serve to dampen mobility of the first residues of the C-terminal region in a manner analogous to what would occur in full length Nsp2. Similar results have been recently reported in a series of eight 100-residue polypeptides corresponding the disordered, prion-like domain of human CPEB3 (Ramírez de Mingo et al. 2020). However, these results may not be general for folded proteins considering that a 2D infrared spectroscopy characterization of His-tagged myoglobin reported an overall decrease in ps dynamics (Thielges et al. 2011) and may affect biological function (Majorek et al. 2014).

An internal proteolytic cleavage seems to impact protein dynamics strongly; in particular on μs-ms timescales its effect is felt throughout the length of the resulting C-terminal fragment of SARS-CoV-2 Nsp2. The study of the structure and dynamics of wild type human frataxin and a C-terminal truncated form also reported more extensive dynamics changes on slower μs-ms timescales (Faraj et al. 2014).

## Materials and methods

### Bioinformatics

Algorithms IUPred2A and ANCHOR (Mészáros et al. 2018; Erdós and Dosztánzi 2020), which are based on energy estimations, were used to predict the disordered regions in the Nsp2 sequence. Two versions of IUPred2A, best suited for short and long disordered sequences, were used. The ANCHOR algorithm seeks to detect disordered sequences that undergo folding upon binding. Furthermore, the PrDOS (Ishida and Kinoshita 2007) as well as the XLXT and XL3 algorithms from the PONDR^®^ suite (Obradovic et al. 2003) which are neural networks optimized with distinct protein training sets and using different attributes, were also utilized. In all cases, the default threshold values for disordered residues and other program settings were used.

### Sample production and isotopic labeling

To enable inhibitor screening and to uncover conformational preferences and dynamics, we have expressed and purified the ^13^C,^15^N-labeled C-terminal region of Nsp2. The full production methodology of this and other SARS-CoV-2 proteins has been recently published (Altincekic et al. 2021). Briefly, the DNA sequence coding for the segment K_557_EIIFLEGETLPTEVLTEEVVLK-TGDLQPLEQPTSEAVEAPLVGT_601_, from the C-terminus of Nsp2 was synthesized (GenScript, New Jersey, USA) and cloned in plasmid pET45b also coding for an N-terminal hexaHis tag and enterokinase cleavage site or in a pET28a-derived plasmid coding for an N-terminal thioredoxin domain, a hexaHis tag and a TEV-protease cleavage site. These plasmids were transformed in *E. coli* BL21star(DE3) and were expressed in minimal media containing ^15^NH_4_Cl and ^13^C-glucose as the sole sources of nitrogen and carbon, respectively, as previously described (Treviño et al. 2018). The domain was purified using the His tag/Ni^++^ immobilized metal affinity chromatography (IMAC), digested with Enterokinase (NEB Biolabs, MA, USA) or TEV-protease produced in-house using the protocol by Blommel and Fox (2007). Afterwards, the protein was separated from the His tag as the flow-through from IMAC and then subjected to a final ion exchange purification step.

### NMR spectroscopy

Samples for NMR spectroscopy contained 1.2–1.5 mM ^13^C,^15^N-labeled Nsp2 CtDR at pH 6.3 in 5.0 mM Na_2_HPO_4_/NaH_2_PO_4_ buffer with 10.0 mM NaCl, 10% D_2_O and 50 μM sodium trimethylsilypropanesulfonate (DSS) as the internal chemical shift reference. These conditions were chosen as an optimal compromise between the low pH and low ionic strength conditions that afford the best quality NMR spectra and physiological conditions. Three series of spectra were recorded at 5 °C: (1) one with an N-terminal His tag whose sequence is MAHHHHHHGTGTGSNDDDD-K, (2) without the His tag and containing an internal cleavage between K_579_ and T_580_, and (3) without the N-terminal His tag and without the internal cleavage. Based on the sequence and using the “Peptide Cutter Tool” in the Expasy website (https://web.expasy.org), trypsin may have caused the adventitious cleavage between K_579_ and T_580_. However, it is more likely to have resulted from the intrinsic broad specificity of enterokinase (Chio et al. 2001).

In contrast to cryo-EM which typically employs much colder temperature, i.e. liquid ethane (− 90 °C), NMR spectroscopy can be applied at near physiological temperatures. Here, we chose to use 5 °C instead of the physiological temperature of 37 °C to reduce exchange of the HN groups, allowing more protein residues to be studied. Moreover, previous studies in our laboratory (Ramírez de Mingo et al. 2020) and others (Abyzov et al. 2016) have shown that whereas slightly higher populations and modestly more rigidity is observed at 5 °C, similar conformational and dynamics trends in IDPs are observed at 5 °C as at higher temperatures.

The series of 2D ^1^H-^15^N HSQC and ^13^C-^15^N CON as well as 3D HNCO, HNCA, CBCAcoNH, and HncocaNH (Pantoja-Uceda and Santoro 2009) spectra were recorded on a Bruker Neo 800 MHz (^1^H) spectrometer equipped with a cryoprobe and *z*-gradients. The NMR spectral parameters are listed in Table 1. The spectra were transformed with Topspin 4.0.8 (Bruker Biospin) and were assigned manually with the aid of the program Sparky (Lee et al. 2015).

### Calculation of conformational chemical shifts

Following the assignment of the experimental ^13^Cα and ^13^CO chemical shifts, the chemical shift values expected for random coil were calculated using the approach of Poulsen and coworkers (Kjaergaard and Poulsen 2011; Kjaergaard et al. 2011) at pH 6.3, 5 °C using the webserver: https://spin.niddk.nih.gov/bax/nmrserver/Poulsen_rc_CS/. These values were subtracted from the experiment chemical shift values to obtain the conformational chemical shift values shown in Fig. 3. To estimate the % population of extended conformations, the conformational chemical shift averaged over five residues was divided by the value of − 1.54 ppm for ^13^Cα (the average of − 1.48 (Spera and Bax 1991) and −1.6 ppm (Santiveri et al 2001) and − 2.21 ppm for ^13^CO (Wishart and Skyes 1994) and multiplied by 100.

### Dynamics

A series of 2D ^1^H-^15^N HSQC-based experiments were recorded to determine the {^1^H}-^15^N NOE and R_1_ρ relaxation rates to assess dynamics on the ps/ns timescale as well as potential contributions from slow (µs/ms) exchange. Peak integrals were measured using Topspin 4.0.8. R_1_ρ experiments used a spin lock of 2 kHz, with 8 relaxation delays varying from 8 to 600 ms, and the corresponding relaxation rates were obtained using the program KaleidaGraph (Synergy Software, version 3.6) to fit an exponential equation: *I*(*t*) = *I*o·exp(− *k*·*t*) + *I* $\infty$, where *I*(*t*) is the peak integral at time *t*, *k* is the rate, and *I* $\infty$ is the intensity at infinite time to the data. The R_1_ρ uncertainties reported here are those obtained from the least squares fit. In the case of the {^1^H}-^15^N heteronuclear NOE experiment, two (^1^H-saturated and non-saturated) experiments are interleaved with a ten second recycling delay from which the corresponding {^1^H}-^15^N NOE ratios are obtained. The uncertainties were calculated as the ratio of the noise (estimated as the standard deviation of the integral of several areas lacking peaks) times $\surd 2$ to the peak integral measured without applying the NOE.

## Supplementary Information

Below is the link to the electronic supplementary material.

Supplementary file1 (DOCX 256 kb)

## Abbreviations

CtDR: C-terminal disordered region
IDP: Intrinsically disordered protein
Nsp2: Non-structural protein 2
SARS-CoV-2: Severe acute respiratory syndrome coronavirus 2

## Declarations

### Availability of data and material

The obtained chemical shift data are deposited in the BMRB database under access code 50687. Requests for materials can be addressed to DVL.
