# Supplementary material for: Partial structure, dampened mobility, and modest impact of a His tag in the SARS-CoV-2 Nsp2 C-terminal region
Source: Eur Biophys J. 2021 Oct 11;50(8):1129–37. doi: 10.1007/s00249-021-01575-9 (PMC8503394; doi:10.1007/s00249-021-01575-9)
Supplement: Supplementary file 1 — Supplementary file1 (DOCX 256 kb) [file 249_2021_1575_MOESM1_ESM.docx]

# Supporting Figure 1

# 2D ^1^H-^15^N HSQC NMR Spectra of

# Nsp2-CtDR in 5 mM KPi, 10 mM NaCl, pH 6.3, 5ºC

#
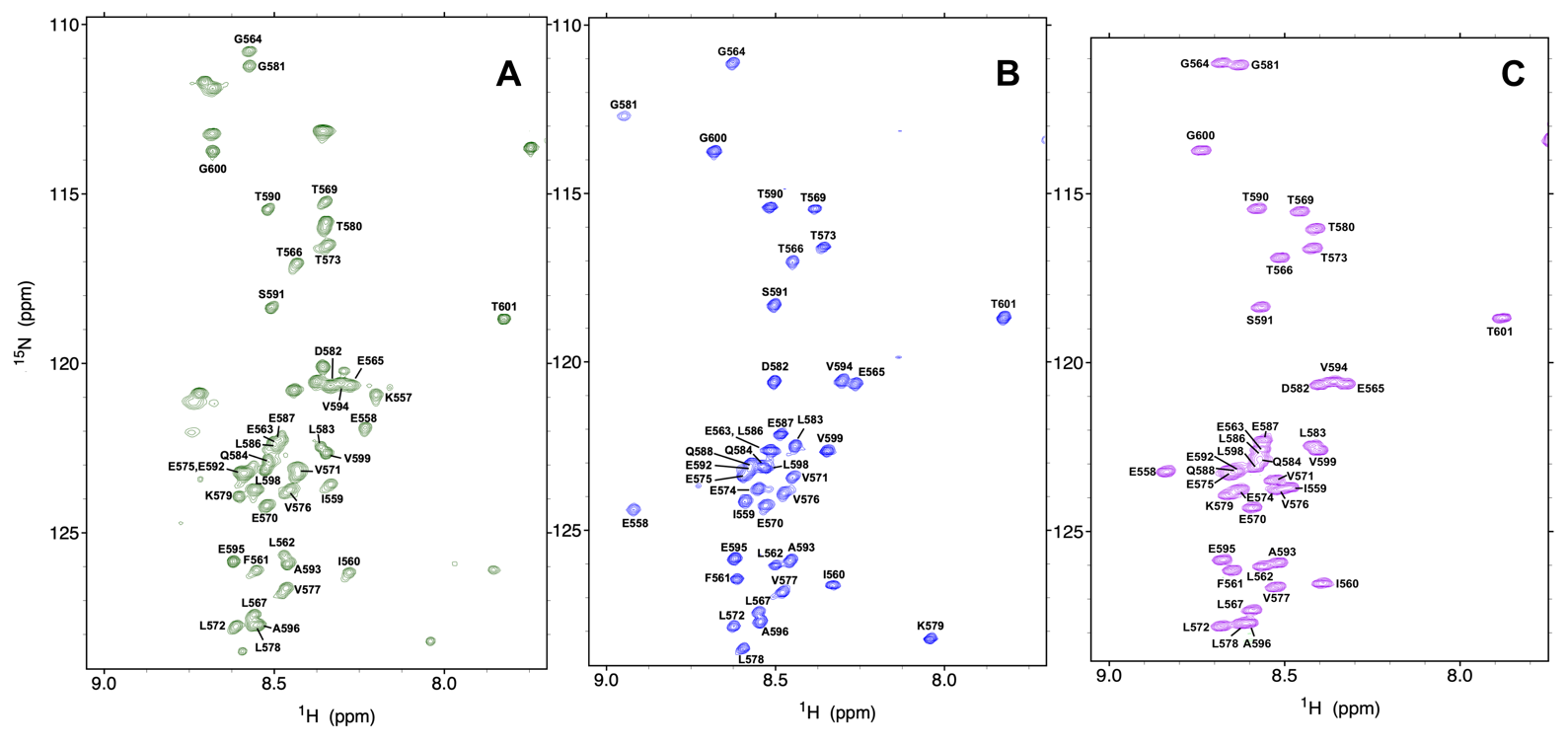


# A. Prior to cleavage of the His tag. Peaks are shown in green and residues are labeled according to their numbering in the full length Nsp2 protein. Note that peaks arising from the N-terminal His tag are not labeled: The His tag sequence is: MAHHHHHHGTGTGSNDDDD-K

# B. Following His tag cleavage; signals are shown in blue. Here the polypeptide contains an additional break between K_579_ and T_580_.

# C. Without the His tag and without the cleavage between K579 and T580. Peaks are colored purple. Due to spectral window optimization, the y-axis of this spectrum is slightly shorter, but the scale is the same.
